# Spatial distribution and associated factors of severe malnutrition among under-five children in Ethiopia: *further analysis of 2019 mini EDHS*

**DOI:** 10.1186/s12889-023-15639-2

**Published:** 2023-04-28

**Authors:** Daniel Gashaneh Belay, Dagmawi Chilot, Adugnaw Zeleke Alem, Fantu Mamo Aragaw, Melaku Hunie Asratie

**Affiliations:** 1 Department of Human Anatomy, School of Medicine, College of Medicine and Health Sciences, University of Gondar, Gondar, Ethiopia; 2 Department of Epidemiology and Biostatistics, Institute of Public Health, College of Medicine and Health Sciences, University of Gondar, Gondar, Ethiopia; 3 Addis Ababa University, College of Health Sciences, Center for Innovative Drug Development and Therapeutic Trials for Africa (CDT-Africa), Addis Ababa, Ethiopia; 4 Department of Human Physiology, University of Gondar, College of Medicine and Health Science, School of Medicine, Gondar, Ethiopia; 5 Department of Women’s and Family Health, School of Midwifery, College of Medicine and Health Sciences, University of Gondar, Gondar, Ethiopia

**Keywords:** Severe, Malnutrition, Inequality, Spatial, Ethiopia

## Abstract

**Background:**

Malnutrition is both a significant cause and a result of poverty and deprivation. In developing nations, child malnutrition is still the main public health issue. Severe malnutrition affects every system of the body and leads to medical instability. The assessment of the burden of severe malnutrition is important for ready-to-use therapeutic foods and preparing therapy for these conditions. Therefore, this study aimed to assess the prevalence and spatial distribution of severe malnutrition and the factors associated with it.

**Methods:**

Data from the 2019 Mini-EDHS (Ethiopian Demographic and Health Surveys) with stratified sampling techniques were used. The data were weighted using sample weight to restore the data's representativeness and provide accurate statistical estimations. A total of 5,006 weighted samples of children under the age of five were used to analyze the study. A multilevel binary logistic regression model was built, and a cutoff P-value of 0.05 was used. The wag staff normalized concentration index and curve as well as spatial analysis were used.

**Results:**

The prevalence of severe malnutrition practice among under five years children in Ethiopia was 14.89% (95%CI: 13.93%, 15.91%), and ranges from 4.58% in Addis Ababa to 25.81% in the Afar region. Women with secondary and above education status as compared to uneducated [AOR = 0.17; 95%CI;[0.06, 0.48], high community women's education as compared to low [AOR = 0.54; 95%CI; 0.36, 0.78], women from richest household as compared to poorest [AOR = 0.63; 95%CI; 0.26, 0.94] and living in Oromia region as compared to Tigray [AOR = 0.33: 95%CI; 0.15, 0.74] were preventive factors. Whereas children 24–59 months of age as compared to under six months [AOR = 1.62; 95%CI; 1.50, 1.75], and being multiple births as compared to single [AOR = 5.34; 95%CI; 1.36,2 1.01] have significant risk factors for severe malnutrition. There was a pro-poor distribution of severe malnutrition among under-five children in Ethiopia with a concentration index of -0.23 [95%CI: -0.27, -0.19]. Severe malnutrition has significant spatial variation over regions in the country where the entire Afar, Eastern Amhara, Southern, and eastern Tigray regions were severely affected (RR = 1.72, *P*-value < 0.01).

**Conclusion and recommendations:**

The prevalence of severe malnutrition in Ethiopia is relatively high as compared to other studies and most of them were severe chronic malnutrition. Having an educated mother/caregiver, and living in a cluster with high community women's education were preventive factors for severe malnutrition in children. Whereas having an unmarried mother/caregiver, old age of the child, plurality of birth, and having double children in the family have a positive association with it. Moreover, it was disproportionately concentrated in poor households (pro-poor distribution).

The spatial distribution of childhood severe malnutrition was not random. Regions like Tigray, Afar, Eastern parts of Amhara, and Somalia regions should be considered priority areas for nutritional interventions for reducing severe malnutrition. Equity-focused nutritional interventions could be needed to curb the wealth-related inequalities of childhood severe malnutrition.

## Background

Malnutrition is a consequence of the consumption of dietary nutrients either insufficiently or exclusively by especially children [1]. It causes about half of all child deaths globally and significantly hinders the growth of children who survive [2]. Malnutrition at the early stages of life can increase the risk of infections, morbidity, and mortality together with decreased mental and cognitive development [3, 4]. In addition, it lowers work productivity and academic achievement and increases the chance of developing chronic diseases later in life [5].

Worldwide, the prevalence of different forms of malnutrition such as stunting (height-for-age), wasting (weight-for-height), and underweight (weight-for-age) in under-five children are still high [6, 7]. In 2020, nearly 149.2 million children under 5 suffer from stunting, 45.4 million children under 5 were wasted globally [8].

Child malnutrition continues to be the leading public health problem in developing countries [9, 10]. Africa is unlikely to meet the world health Organization (WHO) target of reducing global stunting by 40% in 2025 [11, 12]. The WHO 2021 report shows that the number of children with stunting is declining in all regions of the world except Africa [13]. From this, under five malnutrition is predominantly prevalent in sub-Saharan Africa (SSA) region [9, 10]. A meta-analysis study showed that the prevalence of stunting, wasting, and being underweight in SSA were 32.2%, 7.1%, and 16.3%, respectively, in 2016 [14].

Ethiopia is one of the largest populated nations in sub-Saharan Africa and the country with the fifth-highest rate of under-five mortality worldwide in 2018 [15]. In Ethiopia, malnutrition is a leading cause of child illness and death [16]. The prevalence of stunting, being underweight, and wasting were 38.3%, 23.3%, and 10.1%, respectively in Ethiopia [17].

Severe malnutrition is, defined as where the respective anthropometric measurements z-scores are below -3 SD [18]. Children with severe malnutrition have an increased risk of serious illness and death, primarily from acute infectious diseases [19]. The assessment of the burden of severe malnutrition is important for ready-to-use therapeutic foods and preparing therapy for these conditions. Therefore, the purpose of this study was to explore the spatial distributions and to identify the wealth-related inequalities and other variables associated with severe malnutrition.

## Methods

### Study setting, and period

The recent mini Ethiopian Demographic and Health Survey (EDHS, 2019) which was conducted from March 21, 2019, to June 28, 2019, was used to conduct this study [20]. Ethiopia is an East African country located 3^0^ -14^0^ N and 33^0^ – 48^0^E with 1.1 million Sq. km coverage. It is the second most populous country in Africa and is federally decentralized into nine regions and two city administrations [21].

### Source and study population

All under-five children preceding five years of the survey period in Ethiopia were the source population. Whereas, under-five children preceding five years of the survey period in the selected Enumeration Areas (EAs) were our study population. For this study, mothers who had more than one kid, the questionnaires and anthropometric measurements were taken about their most recent child during the two years before the study [22]. The study excludes children who were not weighed or measured, and whose weight and height values were not recorded [23]. Finally, the unweighted 5,121 samples (5,006 weighted samples) were used.

### Sampling technique

A stratified two-stage cluster sampling method was utilized. Every eleven regions were stratified by dividing them into urban and rural areas. In total, 21 sampling strata have been created. Enumeration Areas (EAs) were the sampling units for the first stage of sampling. To ensure that survey precision was comparable across regions, sample allocation was done through an equal allocation where 25 EAs were selected from eight regions. However, 35 EAs were selected from each of the three larger regions: Amhara, Oromia, and the Southern Nations, Nationalities, and Peoples’ Region (SNNPR).In the first stage, a total of 305 EAs (93 in urban areas and 212 in rural areas) were selected with probability proportional to EA size (based on the 2019 EPHC frame) [21, 24].

### Study variables

The outcome variable of this study was severe malnutrition among under-five children. For this study children classified under severe malnutrition, if she/he have either of the three severe malnutrition i.e. severely stunted (height-for-age), severely wasted (weight-for-height), and severely underweight (weight-for-age), with z-score below -3 standard deviations (SD) on the respective anthropometric calculations. The z-scores were calculated using software based on the WHO Anthro program and the macros for statistical packages [21].

The individual-level factors include socio-demographic and socio-economic characteristics such as; the age of the mother, head of the household, marital status, maternal education, drinking water source, and household wealth status were included. Child-related factors such as the sex and age of the child, the plurality of birth, birth order, and the number of under five in the family are all taken. Health service utilization-related factors such as place of delivery and ANC visit were also considered. The community-level factors include; community level women's education, place of residence, and region were considered. The percentage of women with at least a primary education was another way to gauge the education of women at the community level. It was coded as "0" for low (communities with less than 50% of women having at least a primary education) and "1" for high (communities with more than 50% of women having at least a primary education) (at cluster level) [25].

### Data source, processing, and analysis

The DHS kid's records (KR) datasets were obtained in STATA format from the most recent mini-2019 EDHS. For the analysis, the data was cleaned, combined, and coded to yield useful variables. The data were then weighted using sampling weight for probability sampling and non-response to restore the representativeness. To produce both descriptive and analytical statistics, STATA 14 was employed.

### Model building for multi-level analysis

Given the hierarchical nature of the DHS data, the assumptions of the conventional logistic regression model may not hold. A multilevel regression analysis is therefore required. This led to the fitting of four modes, the first of which was employed to gauge the extent of severe malnutrition variation within the cluster. The second model regresses individual-level variables, while third model uses community-level factors with the outcome variable. The final model jointly matched variables at the personal and community levels with the severe malnutrition (Model 4). Model comparisons were made using the deviance test, and the model with the lowest deviance value (model 4) was chosen as the best-fit model.

### Parameter estimation method

The generalized linear mixed model (GLMM) was employed for this study, in which the linear predictor comprises both random and fixed effect analyses. The relationships and strength between severe malnutrition and independent variables were shown using adjusted odds ratios and 95% confidence intervals in the fixed effects measure of association [26–28].

$$Log \left(\frac{\pi ij}{1-\pi ij}\right)=\beta o+ \beta 1xij+ \beta 2xij+\dots uj+eij$$

Where, $\pi ij$: the probability of having severe malnutrition, $1-\pi ij$: the probability of not having severe malnutrition, $\beta$ 1xij are individual and community level variables for the i^th^ individual in group j. The ß’s are fixed coefficients indicating a unit increase in X can cause a ß unit increase in the probability of severe malnutrition. While the ß0 is the intercept that is the effect on severe malnutrition when the effect of all explanatory variables is absent. The uj shows the random effect (effect of the cluster on the mother’s decision to provide severe malnutritio) for the j^th^ cluster. The clustered data nature and the within and between cluster variations were taken into account assuming each cluster has a fixed coefficient (β) and a different intercept (β0) [26, 28, 29].

The measure of variation or random effects was estimated by the median odds ratio (MOR), Intra Class Correlation Coefficient (ICC), and Proportional Change in Variance (PCV).

The ICC which reveals the variation of severe malnutrition between clusters is calculated as; $ICC=\frac{VA}{VA+3.29}*100\%$. Based on this; there were 18% variations of severe malnutrition due to cluster differences among under-five children. The MOR is defined as the median value of the odds ratio of severe malnutrition between the area at the highest risk and the area at the lowest risk when randomly picking out two clusters. MOR = exp.[√(2 × VA) × 0.6745], or ${{\mathrm{MOR}=e}^{0.95}}^{\sqrt{VA}}$ [26–28]. In our study, the median odds ratio between the higher and lower-risk areas of severe malnutrition among clusters was 2.25 [95%CI:2.18, 2.33] in the null model. The PCV reveals the variation in severe malnutrition among children under five explained by factors. The PCV is calculated as; $PCV=\frac{Vnull-VA}{V null}*100\%$ where; Vnull = variance of the initial model, and VA = variance of the model with more terms [26–28]. Moreover, in this study, about 55.56% of the variation in severe malnutrition in under five children was explained by the final model (model four). Likelihood and deviance were used for model comparison and the model with the highest likelihood and the lowest deviance (model 4) was considered the best fit model. There was no multicollinearity between independent variables in all models based on the Variance Inflation Factors (VIF) results (Table 1).

**Table 1:** Parameters and model fit statistics for multilevel regression analysis models

Parameters	Null model	Model 2	Model 3	Model 4
Cluster level Variance	0.72	0.58	0.54	0.34
ICC	0.18	0.15	0.14	0.09
MOR	2.25[2.18, 2.33]	2.05[1.99, 2.11]	2.01[1.96, 2.06]	1.73[1.68,1.78]
PCV	Reference	19.44%	25%	55.56%
**Model fitness**				
Likelihoods	-2025	-573	-1989	-567
Deviance	40,50	1146	39,78	1134
Mean VIF	–-	1.12	1.87	1.45

*ICC* Inter cluster correlation coefficient, *MOR* Median odds ratio, *PCV* proportional change in variance, *VIF* Variance Inflation Factors

### Wealth-related inequalities of severe malnutrition

To examine the socioeconomic inequalities of severe malnutrition, the concentration index and graph approach were used [30, 31]. The concentration curve was applied to identify whether there was socioeconomic inequality in severe malnutrition or if it was more pronounced in one group [31, 32].

The concentration curve would be a 45^0^ line indicating the absence of inequity while, the concentration curve laying above and below the equality line (45^0^) indicated that severe malnutrition is disproportionately concentrated between poor and rich, respectively [33].

The concentration index is twice the area between the concentration curve and the diagonal line [32, 34]. It ranges from − 1 to + 1 and the sign indicates the direction of the relationship between the health variable (severe malnutrition) and the distribution of living standards (wealth status).

**Fig. 1:**
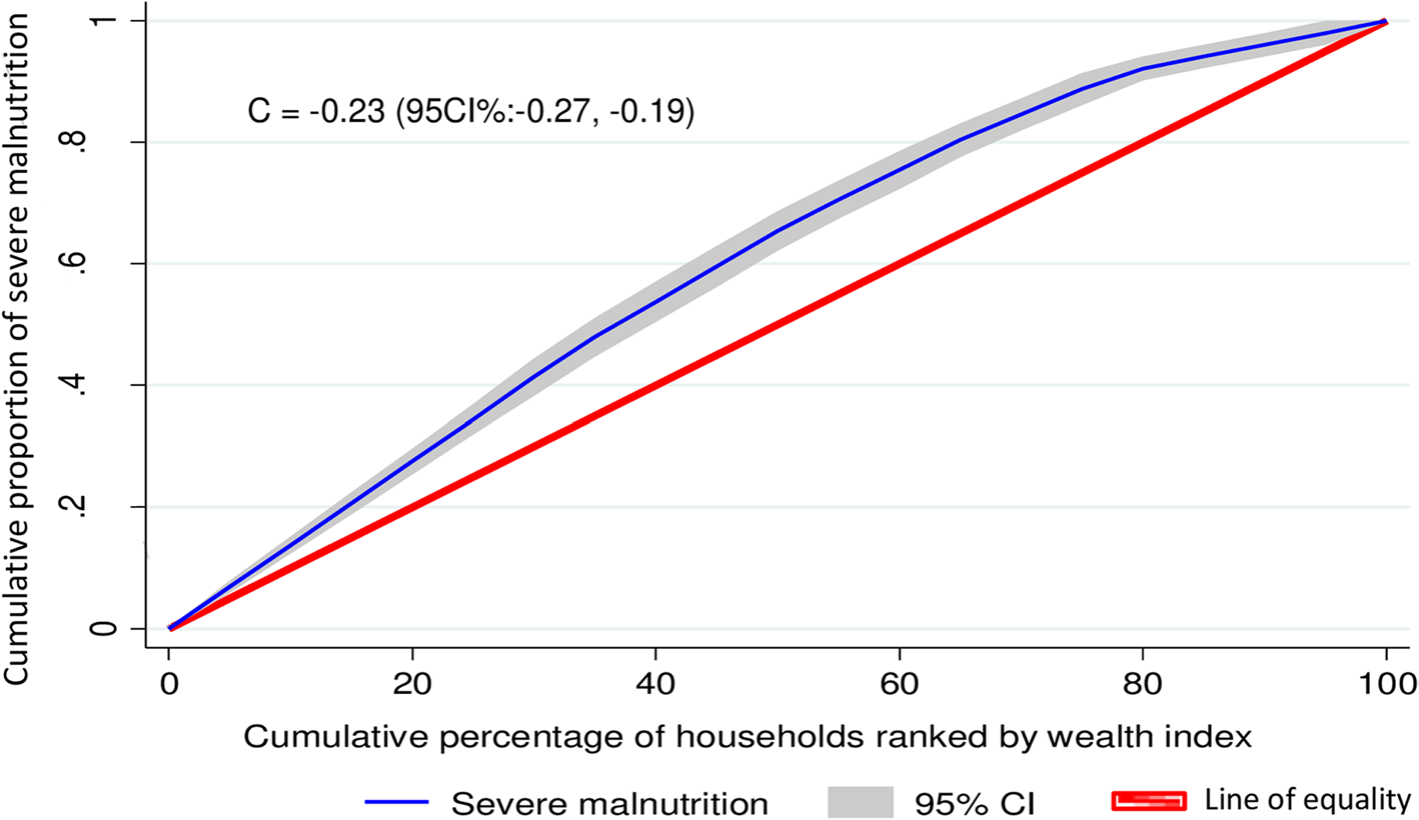
Wealth-related inequalities of severe malnutrition among under-five children in Ethiopia

In our study, there was a pro-poor distribution of severe malnutrition among under-five children in Ethiopia with a concentration index of -0.23 [95%CI: -0.27, -0.19] (Fig. 1).

### Spatial analysis of severe malnutrition among under-five children in Ethiopia

To assess the spatial distribution of severe malnutrition among under-five children in Ethiopia, Global Moran’s I statistic spatial autocorrelation measure was used [35]. Whereas a spherical semivariogram ordinary kriging type spatial interpolation technique was used to predict severe malnutrition among under-five children in Ethiopia for unsampled areas based on sampled clusters. The proportion of severe malnutrition among under-five children in each cluster was taken as an input for spatial prediction.

Using Kuldorff's SaTScan version 9.6 software, Bernoulli-based model spatial scan statistics were used to pinpoint the locations of statistically significant clusters for severe malnutrition among children under the age of five [36]. The scanning window that moves across the study area in which children who had severe malnutrition were taken as cases and those children who had not severe malnutrition were taken as controls to fit the Poisson model.

## Results

### Mothers or caregivers and children socio demographic characteristics.

A total weighted sample of 5,006 under-five children was included in this study. More than half (54.07%) of mothers of children were in the age group of 25–34 years with a median age of 28 (IQR: 8) years. More than half of women (53.72%) had no formal education. Moreover, half (52.06%) of children were delivered at home.

Nearly three-fifths (59.83%) of the children were found in the age group from 24–59 months with a median age of 28 (IQR: 30) months. Almost all (97.82%) of the child are single birth. Three-fourths (74.96%) of the children lived in rural and 40% of the children are from the Oromia region (Table 2).

**Table 2:** Socio-demographic characteristics of the mothers/caregivers and the children in a study of severe malnutrition and associated factors among under-five children in Ethiopia

Variables	Categories	Frequency	Percentage	Case prevalence
**Socio-demographic characteristics**				
Age of women (years)	15–24	1,146	22.89	11.98
	25–34	2,707	54.07	15.99
	36–49	1,154	23.05	15.20
Sex of household head	Male	4,356	87	14.86
	Female	651	13	15.11
Educational attainment of women	No education	2,690	53.72	19.65
	Primary education	1,767	35.3	11.05
	Secondary&above	549	10.97	3.93
Marital status of the mother	Married	4,718	94.25	14.92
	Not married	288	5.75	16.33
Wealth index	Poorest	1,160	23.17	21.45
	Poorer	1,104	22.05	16.85
	Middle	938	18.74	15.41
	Richer	883	17.63	12.74
	Richest	922	18.41	5.82
Water source	Improved	3,057	61.06	14.36
	Unimproved	1,950	38.94	15.71
**Child related characteristics** and** health service utilization of the mothers**				
Birth order	< = 3^rd^	2,686	53.65	12.39
	> 3^rd^	2,320	46.35	17.78
Sex of child	Male	2,544	50.81	17.85
	Female	2,463	49.19	11.83
Age of child	0–5	531	10.6	7.7
	6–23	1,480	29.56	11.65
	24–59	2,995	59.83	17.76
Plurality of birth	Single	4,897	97.82	14.75
	Multiple	109	2.18	21.25
Number of under 5 children	One	1,973	39.41	12.22
	Two	2,332	46.58	16.07
	Three and more	701	14.01	18.46
Place of delivery	Home	2,606	52.06	18.03
	Health institution	2,400	47.94	11.48
ANC visit	No ANC visit	900	24.81	16.14
	At least one visit	2,728	75.19	12.91
**Community level variables**				
Residence	Urban	1,254	25.04	7.82
	Rural	3,753	74.96	17.25
Regions	Tigray	352	7.03	17.06
	Afar	75	1.51	25.81
	Amhara	961	19.2	15.88
	Oromia	1,994	39.83	13.30
	Somali	345	6.9	20.01
	B/Gumuz	61	1.21	21.92
	SNNPE	1,018	20.33	15.10
	Gambela	22	0.43	6.99
	Harari	14	0.29	15.24
	Addis Ababa	138	2.76	4.58
	Dire Dawa	26	0.52	7.81
Community-women education	Low	2,243	44.8	21.01
	high	2,764	55.2	9.93

### Prevalence of severe malnutrition among under-five children in Ethiopia

**Fig. 2:**
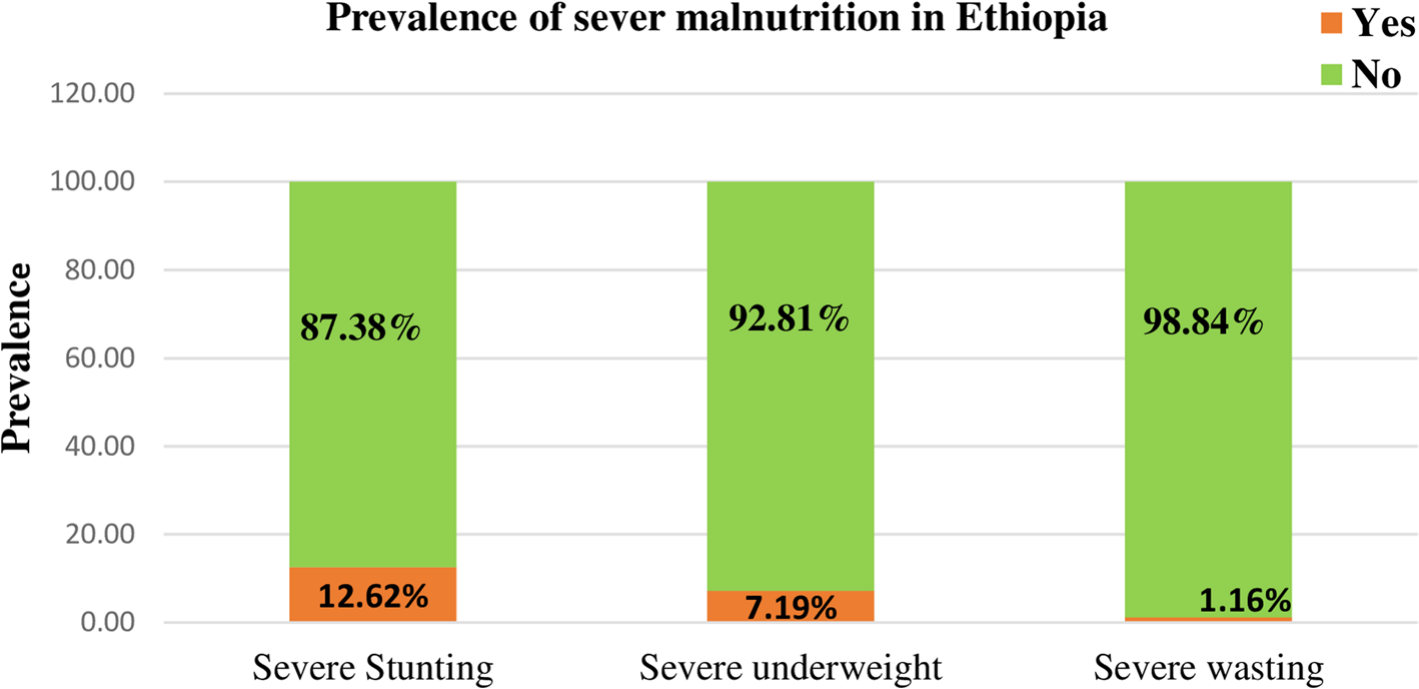
The bar graph shows the prevalence of severe malnutrition among under-five children in Ethiopia

The prevalence of severe malnutrition among under-five children in Ethiopia was 14.89% (95%CI: 13.93%, 15.91%), and ranges from 4.58% in Addis Ababa to 25.81% in Afar. Of which, most of them were chronic severe malnutrition i.e. severe stunting (12.62%), others were severely underweight (7.19%) and severe wasting (1.16%) (Fig. 2).

### Multi-level analysis of the determinants of severe malnutrition among under-five children in Ethiopia

The plurality of births, region, age of the child, marital status of the mother/caregiver, wealth index of the household, and education status of women were all significant variables in the chosen model (model 4).

Secondary and above-educated women were 83% less likely to have children with severe malnutrition than women with no formal education [AOR = 0.17; 95%CI;[0.06, 0.48]. Moreover, children who were from high-community education clusters were 46% less likely to have severe malnutrition than those from low-community women's education [AOR = 0.54; 95%CI; 0.36, 0.78].

The odds of having severe malnutrition among children from not currently married mothers were 2.69 times higher as compared to a child from a married one [AOR = 2.69; 95%CI; 1.35, 5.36].

Children who come from the richest wealth family were 37% less likely to have severe malnutrition than the poorest family [AOR = 0.63; 95%CI; 0.26, 0.94].

Being multiple births and having two under-five children in the family were 5 and 2 times more likely to have severe malnutrition than those who were singleton birth and single child [AOR = 5.34; 95%CI; 1.36,2 1.01] and [AOR = 2.01; 95%CI; 1.35, 2.99] respectively.

The odds of severe malnutrition among children aged 6–23 and 24–59 months were 1.93 and 1.62 times higher than children who were found 0–5 months age [AOR = 1.93; 95%CI; 1.27, 2.93] and [AOR = 1.62; 95%CI; 1.50, 1.75] respectively. Children who live in the Oromia region were 66% less likely to have severe malnutrition than those who live in Tigray [AOR = 0.33: 95%CI; 0.15, 0.74] (Table 3).

**Table 3:** Multilevel analysis of factors associated with severe malnutrition among children age 0–59 months in Ethiopia

Variables	Categories	^a^Model 2	Model 3	Model 4
		AOR [95% CI]	AOR [95% CI]	AOR [95% CI]
**Socio-demographic characteristics**				
Age of women (years)	15–19	1.00	------------	1.00
	20–35	0.66 [0.41, 1.06]	------------	0.67 [0.42, 1.08]
	36–49	0.77 [0.41, 1.46]	------------	0.78 [0.42, 1.47]
Sex of household head	Male	1.00	------------	1.00
	Female	0.91 [0.52, 1.61]	------------	0.84[0.47, 1.51]
Educational attainment of women	No education	1.00	------------	1.00
	Primary education	0.68[0.45, 1.02]	------------	0.73[0.48, 1.12]
	Secondary&above	0.16 [0.06, 0.45]**	------------	0.17 [0.06,0.48]***
Marital status of the mother	Married	1.00	------------	1.00
	Not married	2.75 [1.38, 5.49]***	------------	2.69 [1.35, 5.36]**
Wealth index	Poorest	1.00	------------	1.00
	Poorer	1.44 [0.87, 2.40]	------------	1.63 [0.96, 2.75]
	Middle	1.18 [0.67, 2.10]	------------	1.33 [0.74, 2.38]
	Richer	1.66 [0.92, 3.03]	------------	1.85 [0.99, 3.44]
	Richest	0.60 [0.28, 0.92]*	------------	0.63 [0.26, 0.94]*
Water source	Improved	1.00		1.00
	Unimproved	1.04 [0.72, 1.55]		1.03 [0.70, 1.52]
**Child related characteristics** and** health service utilization of the mothers**				
Birth order	< = 3^th^	1.00	------------	1.00
	> 3^th^	1.28 [0.79, 2.01]	------------	1.25 [0.79, 1.98]
Sex of child	Male	1.00	------------	1.00
	Female	0.28 [0.19, 0.41]*	------------	0.28 [0.20, 0.40]*
Age of child	0–5	1.00	------------	1.00
	6–23	1.94 [1.28, 2.95]*	------------	1.93 [1.27, 2.93]*
	24–59	1.35 [1.26, 1.46]**	------------	1.62 [1.50, 1.75]**
Plurality of birth	Single	1.00	------------	1.00
	Multiple	5.14 [1.30, 20.34]*	------------	5.34 [1.36,2 1.01]*
Number of under 5 children	One	1.00	------------	1.00
	Two	2.01 [1.35, 3.01]		2.01 [1.35, 2.99]*
	Three and more	1.33 [0.76, 2.43]	------------	1.43 [0.77, 2.63]
Place of delivery	Home	1.00	------------	1.00
	Health institution	0.74 [0.49, 1.11]	------------	0.72 [0.48, 1.09]
ANC visit	No ANC visit	1.00	------------	1.00
	At least one visit	1.12 [0.74, 1.67]	------------	1.31[0.67, 1.56]
**Community level variables**				
Residence	Urban	------------	1.00	1.00
	Rural	------------	2.04[1.38, 3.04]**	0.93 [0.47, 1.83]
Regions	Tigray	------------	1.00	1.00
	Afar	------------	1.37 [0.67, 2.83]	1.11 [0.29,4.23]
	Amhara	------------	0.65 [0.39, 1.06]	0.81 [0.37, 1.80]
	Oromia	------------	0.64 [0.41, 0.99]*	0.33 [0.15, 0.74]*
	Somali		0.85 [0.48, 1.48]	0.63 [0.21, 1.79]
	B/Gumuz	------------	1.33 [0.60, 2.93]	0.58 [0.04, 9.41]
	SNNPE	------------	0.84 [0.52, 1.35]	0.47 [0.21,1.07]
	Gambella	------------	0.49 [0.08, 2.87]	0.58 [0.03, 9.41]
	Harari	------------	1.01 [0.21, 4.92]	0.85 [0.03,19.51]
	Addis Ababa		0.48 [0.18, 1.28]	0.38 [0.05, 2.83]
	Dire Dawa		0.43 [0.09, 2.04]	0.50 [0.03, 7.54]
Community-women education	Low	------------	1.00	1.00
	high	------------	0.52 [0.39, 0.72]*	0.54 [0.36, 0.78]**

*AOR* Adjusted Odds Ratio; *CI* Confidence Interval, *ANC* Ante Natal Care

^*^  *P*-value < 0.05, **P value < 0.01, ***P value < 0.001

^a^Model 1(null model) = the model which contains only with the dependent variable and values expressed in Table 4

### Spatial analysis of severe malnutrition among under-five children in Ethiopia

**Fig. 3:**
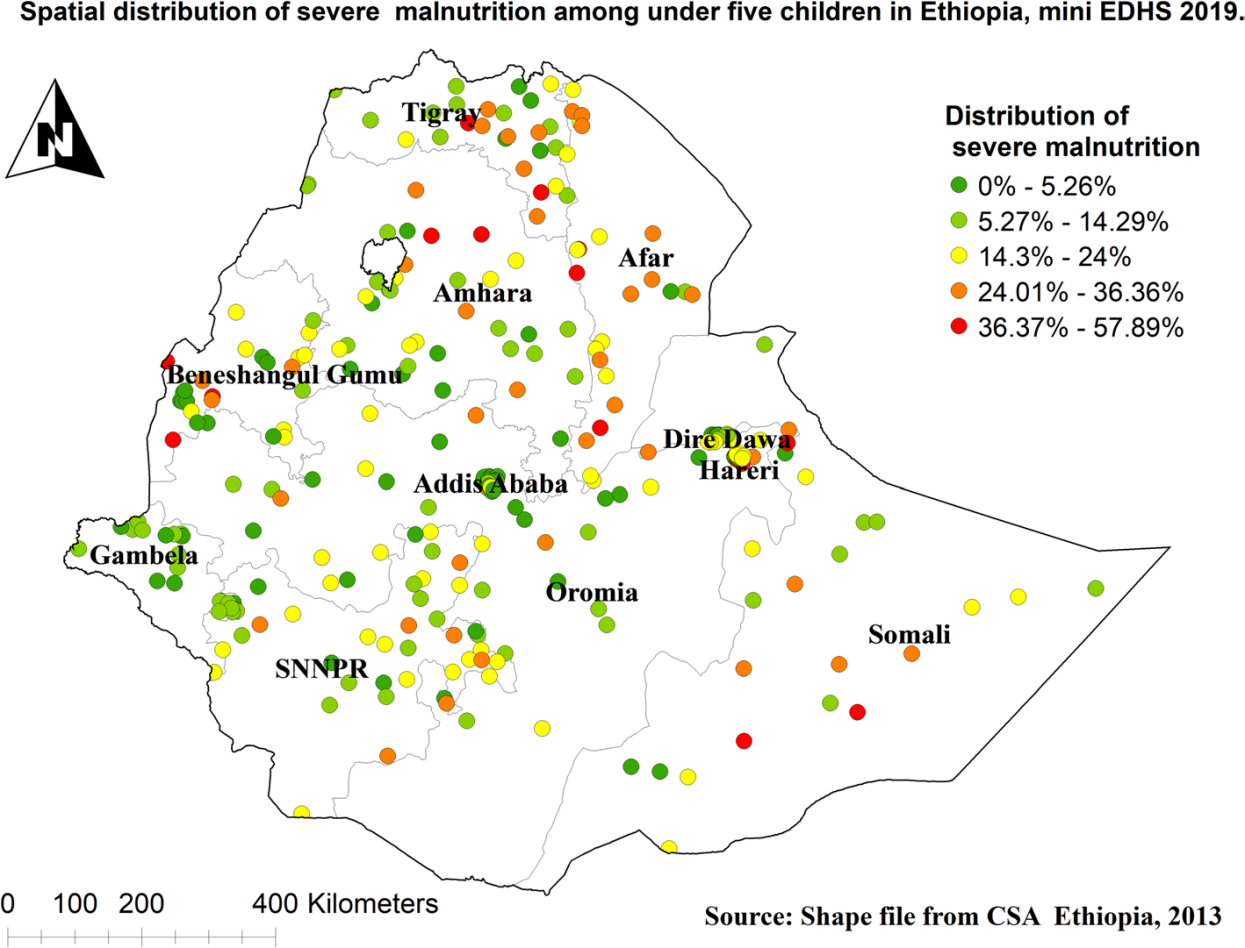
Spatial distribution of severe malnutrition among under-five children in Ethiopia

The spatial autocorrelation result of severe malnutrition among under-five children in Ethiopia showed significant spatial variation over regions in the country and was found to be clustered with Global Moran's Index value: 0.384988 with (*p* < 0.01)**.** Severe malnutrition had more prevalent in Afar, Amhara and Tigray regions and ranges from 36.37% to 57.89% (Fig. 3).

**Fig. 4:**
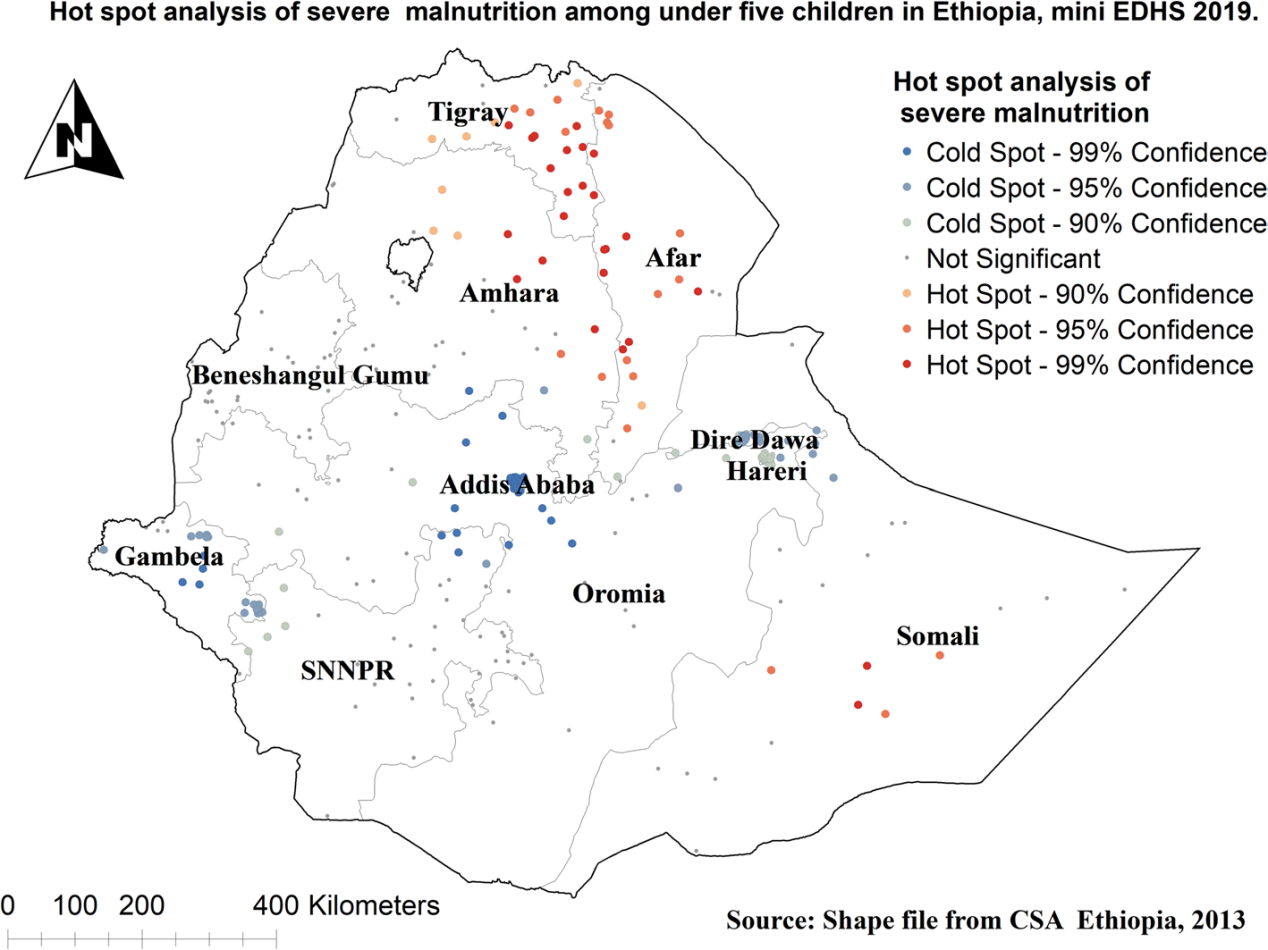
Hotspot analysis of severe malnutrition among under-five children in Ethiopia

The incremental autocorrelation result showed that there is one significant peak distance at 227.328 km; 7.09 (distances; Z-score) for severe malnutrition were most pronounced using 10 distance bands. Severe malnutrition is more common and hot spotted in Tigray, Afar, Eastern parts of Amhara, and Somalia regions, and ranges from 5.26% to 57.89% (Fig. 4).

**Fig. 5:**
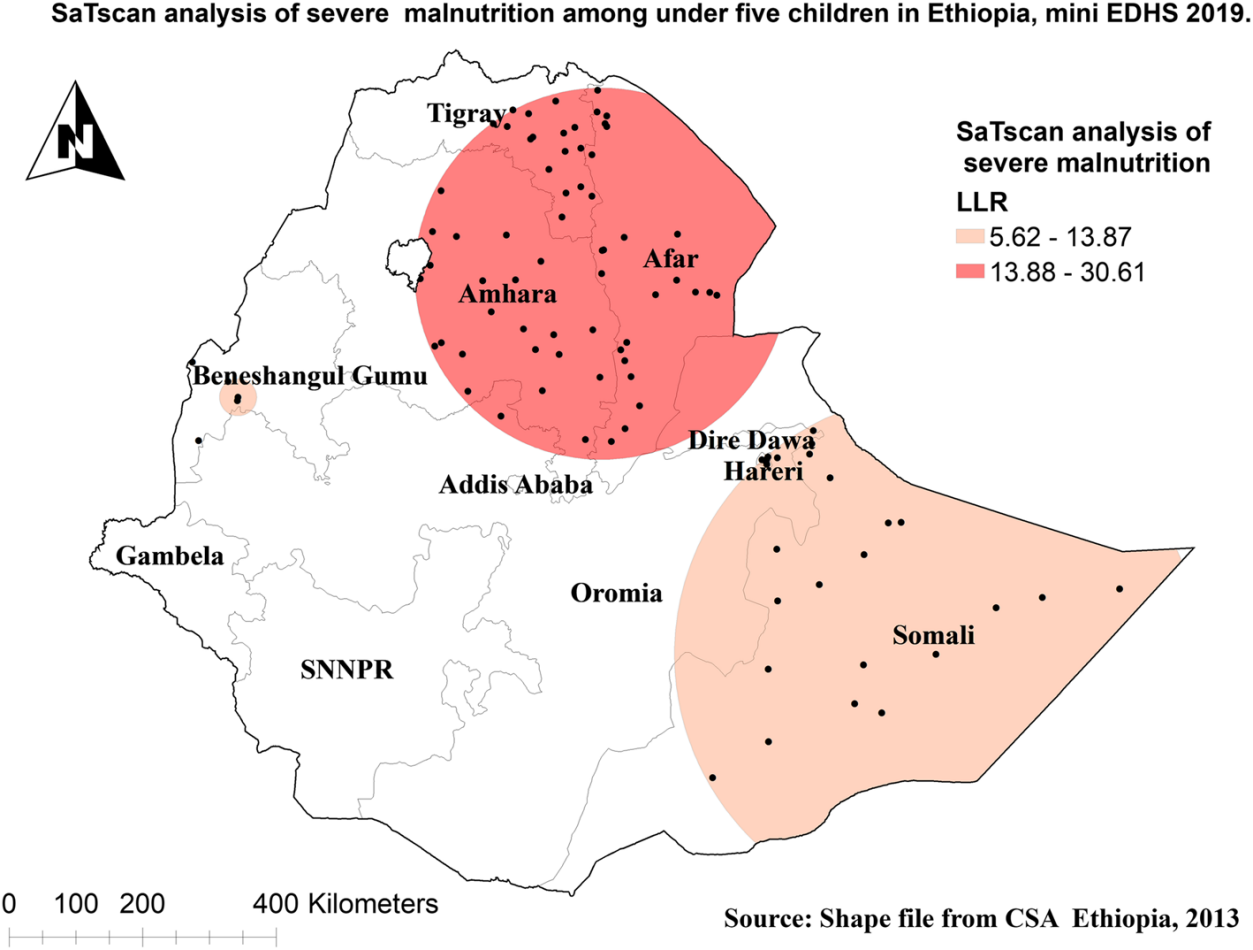
Sat Scan analysis of severe malnutrition among under-five children in Ethiopia

The SaTscan analysis of severe malnutrition among under-five children in Ethiopia showed that 61 primary clusters and 27 secondary clusters were detected for having severe malnutrition. The primary clusters were centered at 11.818783 N, and 39.955788 E with a 279.39 km radius. These were located in the entire Afar, Eastern Amhara, Southern, and eastern Tigray regions. Children who were found in the primary window were 1.72 times more likely to have severe malnutrition than out in-window regions (RR = 1.72, *P*-value < 0.01) (Table 4 and Fig. 5).

**Table 4:** Significant spatial clusters of severe malnutrition among under-five children in Ethiopia using mini EDHS 2019

Clusters	Coordinate/radius	Population	Cases	RR	LLR	*P*-value
1^ry^ [61]	11.818783 N, 39.955788 E / 279.39 km	1116	271	1.72	25.1	< 0.0001
2^nd^ [27]	6.639662 N, 44.465855 E / 390.28 km	585	142	1.59	11.3	0.003

**Fig. 6:**
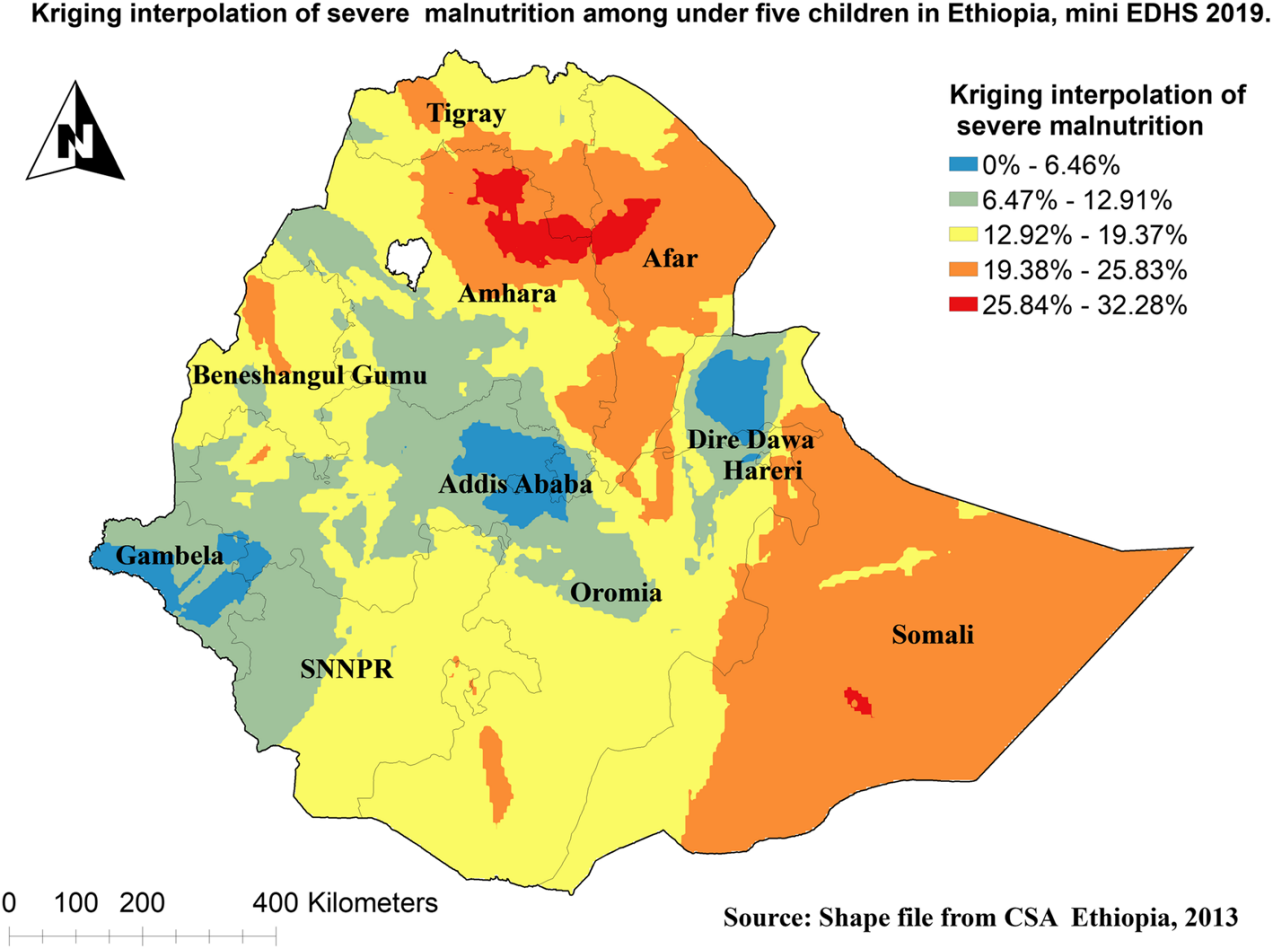
Kriging interpolations of severe malnutrition among under-five children in Ethiopia

The Kriging interpolation methods of predicting severe malnutrition among under-five children in Ethiopia showed that high-risk areas predicted severe malnutrition ranging from 25.84% to 32.28% and are located in Northern and Eastern parts of Amhara, Western parts of Afar, and most parts of the Somali regions. Whereas the lower predicted area was seen in, Addis Ababa, Dire Dawa, and Gambella regions ranging from 0% to 6.46% (Fig. 6).

## Discussions

The world is not on course to meet the targets for malnutrition in 2030. In 2018, globally only 12.2%, 3%, and 3% of 2030 targets for stunting, underweight, and wasting were met respectively [15]. Malnutrition is both a significant cause and a result of poverty and deprivation [15]. Severe malnutrition affects every system of the body and leads to medical instability [37]. In this study, the prevalence of severe malnutrition among under-five children in Ethiopia was 14.89% (95%CI: 13.93%, 15.91%). Similarly, a report from 2018, by UNICEF shows that the prevalence of severe wasting and severe and moderate stunting in Ethiopia were 3% and 38% respectively [15]. A study also showed that the prevalence of severe malnutrition was highest within countries in East Africa [14]. The global rates of severe malnutrition also remain high and around 16.6 million children under 5 were estimated to suffer from severe wasting in 2018 [15]. This high prevalence might be due to population growth, and socio-economic status of the country [38]. Cultural beliefs and knowledge paradigms about under five nutrition might have also influence on child feeding practices [30, 31].

In our study, there was a pro-poor distribution of severe malnutrition among under-five children in Ethiopia. The multilevel analysis result also showed that children who come from the richest wealth family were less likely to have severe malnutrition. In line with a study conducted in 47, developing countries showed that stunting and wasting disproportionately affected the poor [32]. The UNICEF, 2019 report also says that stunting is an accurate reflection of inequality in societies [15]. Moreover, a study showed that minimum acceptable diet (MAD) intake among children was disproportionately concentrated in rich households (pro-rich) [33]. It is expected that children’s from a family of higher income can feed frequent and diversified foods as their families could be more likely to afford to purchase it [34].

In this study women with secondary and above education status have fewer odds of having children with severe malnutrition. Moreover, children who were from high-community women's education clusters were less likely to have severe malnutrition than those from low-community women's education. This could be due to that educated women are more likely to have access to health messages, and can easily comprehend and translate that information into practice and promote optimal child nutrition [25, 33].

The odds of having severe malnutrition among children from not currently married mothers were higher as compared to a child from a married one. Supported by a study that shows children from currently not married women were less likely to have access to MAD [25, 31, 39]. This might be due to that not married mother lack of support from families or communities, which causes poor infant-feeding practices [39, 40].

In this study, multiple births and two under-five children in the family were more likely to have severe malnutrition than their counterparts. A study in Nigeria showed that children of multiple births are more likely to be stunted [41]. Mothers with twins or triplets were twice as likely to choose bottle feeding [42]. In children of multiple births, inadequate breastfeeding and competition for nutritional intake occur more frequently [41].

In this study, the odds of having severe malnutrition becomes increase as the age of the child increased. This is might be due to bottle feeding and toothing becoming more common in this age group [39, 43] which eventually leads to diarrhea, vomiting, and infections [43].

The spatial outputs of this study state that severe malnutrition is more common in Tigray, Afar, Eastern parts of Amhara, and Somalia regions. Moreover, in multilevel analysis children who live in the Oromia region were less likely to have severe malnutrition than those who live in the Tigray region. Other studies also showed that there were spatial variations in malnutrition [44, 45]. This is because these parts of Ethiopia are experienced higher drought frequency than others [46]. Having poor access to healthcare services and feeding practices depending on their way of life in pastoral regions (Afar and Somali) has also contributed [47].

## Conclusion and recommendation

Generally, the prevalence of severe malnutrition in Ethiopia is relatively high as compared to other studies and most of them were severe chronic malnutrition. Having educated women, and living in a cluster with high community women's education were preventive factors for severe malnutrition children. Whereas having an unmarried mother/caregiver, being old age of the child, the plurality of birth, and having 2 under-five children in the family have a positive association with it. Moreover, severe malnutrition among under-five children was disproportionately concentrated in poor households (pro-poor distribution).

The spatial distribution of childhood severe malnutrition was not random. Regions like Tigray, Afar, Eastern parts of Amhara, and Somalia regions should be considered priority areas for nutritional interventions for reducing severe malnutrition. Equity-focused nutritional interventions could be needed to curb the wealth-related inequalities of childhood severe malnutrition.

### Strength and limitation

Some of the strengths of this study include the use of recent nationally representative large sample data, the utilization of wealth-related inequalities, and the spatial distribution analysis. Due to the cross-sectional nature of the data, recall and social desirability bias may be present.

## Data Availability

Data from open databases are accessible to the general population. The website listed below allows access to it https://dhsprogram.com/data/dataset_admin/login_main.cfm?CFID=10818526&CFTOKEN=c131014a480fe56-4E0C6B7F-F551-E6B2-50.
